# Agronomic Strategies to Improve N Efficiency Indices in Organic Durum Wheat Grown in Mediterranean Area

**DOI:** 10.3390/plants10112444

**Published:** 2021-11-12

**Authors:** Federica Carucci, Giuseppe Gatta, Anna Gagliardi, Pasquale De Vita, Simone Bregaglio, Marcella Michela Giuliani

**Affiliations:** 1 Department of Agricultural Sciences, Food, Natural Resources and Engineering (DAFNE), University of Foggia, 71122 Foggia, Italy; federica.carucci@unifg.it (F.C.); giuseppe.gatta@unifg.it (G.G.); anna.gagliardi@unifg.it (A.G.); 2 Council for Agricultural Research and Economics, Research Centre for Cereal and Industrial Crops (CREA-CI), 71122 Foggia, Italy; pasquale.devita@crea.gov.it; 3 Council for Agricultural Research and Economics, Research Centre for Agriculture and Environment (CREA-AA), 40128 Bologna, Italy; simoneugomaria.bregaglio@crea.gov.it

**Keywords:** durum wheat, organic farming, nitrogen, sulfur, selenium, foliar application, NUtE, N uptake

## Abstract

Organic farming systems are often constrained by limited soil nitrogen (N) availability. Here we evaluated the effect of foliar organic N and sulphur (S), and selenium (Se) application on durum wheat, considering N uptake, utilization efficiency (NUtE), grain yield, and protein concentration as target variables. Field trials were conducted in 2018 and 2019 on two old (Cappelli and old Saragolla) and two modern (Marco Aurelio and Nadif) Italian durum wheat varieties. Four organic fertilization strategies were evaluated, i.e., the control (CTR, dry blood meal at sowing), the application of foliar N (CTR + N) and S (CTR + S), and their joint use (CTR + NS). Furthermore, a foliar application of sodium selenate was evaluated. Three factors—variety, fertilization strategies and selenium application—were arranged in a split-split-plot design and tested in two growing seasons. The modern variety Marco Aurelio led to the highest NUtE and grain yield in both seasons. S and N applications had a positive synergic effect, especially under drought conditions, on pre-anthesis N uptake, N translocation, NUtE, and grain yield. Se treatment improved post-anthesis N uptake and NUtE, leading to 17% yield increase in the old variety Cappelli, and to 13% and 14% yield increase in Marco Aurelio and Nadif, mainly attributed to NUtE increase. This study demonstrated that the synergistic effect of foliar applications could improve organic durum wheat yields in Mediterranean environments, especially on modern varieties.

## 1. Introduction

Durum wheat organic systems are often characterized by limited nitrogen (N) availability, due to low soil fertility and the use of organic N fertilizers [1]. Unravelling the real factors causing low soil N availability in organic systems requires additional experimental evidence, given the complex and dynamic transformations of soil organic N. As a consequence, this subject is highly debated in literature, especially in Mediterranean regions: Fagnano et al. [2] reported that low soil organic matter content, low temperatures, and high autumn–spring rainfall can cause deficient levels of available soil N during most wheat crop cycles; on the contrary, Rossini et al. [3] highlighted that soil temperature and water availability during winter can sustain the mineralization activity of the soil microbial community. Another critical aspect in organic systems is the measurement of nitrogen use efficiency (NUE), which is hampered by the complexity of the N cycle, in turn linked to the different organic matter sources and by the limited availability of soil mineral N [1]. As an alternative, several authors have proposed to substitute NUE with the nitrogen utilization efficiency index (NUtE), which is a component of NUE [4]. Higher NUtE has been correlated with higher yield in the modern varieties, which show a significant partitioning of dry matter and N to grain [5,6]. On the other hand, Foulkes et al. [7] suggested that in organic growing systems, old varieties may be more suitable than modern ones, due to their higher efficiency in recovery N from the soil. In any case, all scientists agree that NUtE improvement is largely associated with the choice of the wheat variety, which results as an essential management practice in organic systems.

Furthermore, the main N input in organic systems is constituted by the application of exogenous organic matter in pre-sowing [8,9], without any topdressing or foliar fertilizations. The latter is demonstrated to increase NUE in conventional systems, whereas their effect in organic farming is still not quantified and addressed by a specific experimental study. Sulfur (S) is another key element in durum wheat nutrition, being an essential constituent of enzymes involved in N metabolism as nitrate and nitrite reductase [10,11,12]. S deficiency leads to a decrease in N assimilation, and it has been demonstrated that S addition improved NUE mainly by increasing the soil N recovery in conventional bread wheat [13]. Again, despite many studies having been conducted on the effect of N and S fertilizers on wheat yield and quality under conventional farming [14,15,16,17,18], few studies have investigated the effect of S fertilization on interaction with organic N source on nitrogen efficiency indices. The current Commission Regulation (EC) No 889/2008 lists eligible elements in organic farming, including trace elements as boron (B), cobalt (C), copper (Cu), iron (Fe), manganese (Mn), molybdenum (Mo) and zinc (Zn). Selenium (Se) is still not included in this list, although its action on plants has been studied for almost 70 years [19] and its beneficial effects on plant productivity has been extensively proved [20,21,22,23], including on wheat yields [22]. Other documented positive effects of Se application are the improvement of plant tolerance to drought stress [24] and the increased resistance to fungal infections and invertebrate phloem-feeders [25]. Furthermore, it has been found that foliar Se applications in the form of selenate as an alternative to soil distribution, minimizes the impact on soil microbiology, improving its effectiveness even at low application rate [26]. We tested here for the first time the application of Se in the organic durum wheat system, to evaluate the feasibility of its possible inclusion into the European Organic Production Regulation.

The aim of this work was the evaluation of alternative organic fertilization strategies based on foliar application of N, S and Se on nitrogen efficiency indices of four durum wheat varieties grown under organic management in Mediterranean regions. We conducted a two-year field experiment with the overarching objective of highlighting most promising solutions to support organic durum wheat farmers in the area.

## 2. Results

### 2.1. Growing Season × Variety Effects

Analysis of variance of N-related traits, N efficiency indices, grain yield and grain protein concentration revealed highly significant differences in most considered variables (Table 1).

**Figure 1:**
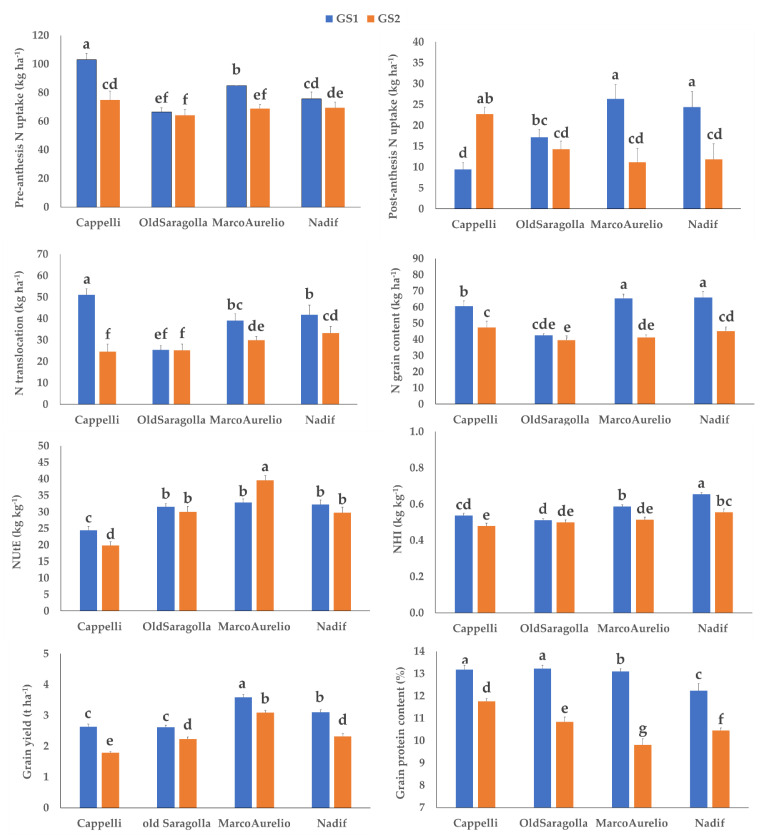
Effect of the interaction growing season × variety on pre-anthesis N uptake (kg ha^−1^), post-anthesis N uptake (kg ha^−1^), N translocation (kg ha^−1^), nitrogen utilization efficiency (NUtE; kg kg^−1^), nitrogen harvest index (NHI; kg kg^−1^), N grain content (kg ha^−1^), grain yield (t ha^−1^) and grain protein concentration (%). GS1, 2017–2018 growing season; GS2, 2018–2019 growing season. Letters indicate significant differences at *p* ≤ 0.05 according to Tukey’s test. Vertical bars indicate standard errors (n = 24).

Cappelli and Marco Aurelio showed significantly lower values of pre-anthesis N uptake in the 2018–2019 growing season (GS2); on the contrary, only Cappelli showed a significant increase of post-anthesis N uptake in GS2, with the two modern varieties showing a marked decrease (Figure 1). N translocation, NHI and N grain content decreased in GS2 in all varieties except in Old Saragolla. The variation of NUtE in the two growing seasons was evident only in Cappelli and Marco Aurelio, with opposing trends: a NUtE decrease in Cappelli and a NUtE increase in Marco Aurelio. Grain yield and grain protein concentration decreased in GS2, the driest season, in all varieties (Figure 1).

### 2.2. Growing Season × Fertilization Strategy Effects

The alternative fertilization strategies differently influenced N parameters, grain yield and grain protein concentration in the two growing seasons (Table 2). In GS2, an evident increase in pre-anthesis N uptake and a slight increase in N translocation were observed only under control plus the combination of S and N foliar applications (CTR + NS). Post-anthesis N uptake decreased under CTR + NS and increased under control plus N foliar application (CTR + N). For NUtE, significant differences between the two growing seasons were observed only under CTR + N and control plus S foliar application (CTR + S) with an opposite trend: in GS2 CTR + N showed lower NUtE values and CTR + S higher respect to 2017–2018 growing season (GS1). Finally, NHI, N grain content, grain yield and grain protein concentration showed lower values in GS2 under all fertilization strategies. Grain yield did not show any significant difference in GS1 across fertilization treatments, whereas in GS2 higher production was obtained under CTR + NS.

### 2.3. Selenium Effects

**Figure 2:**
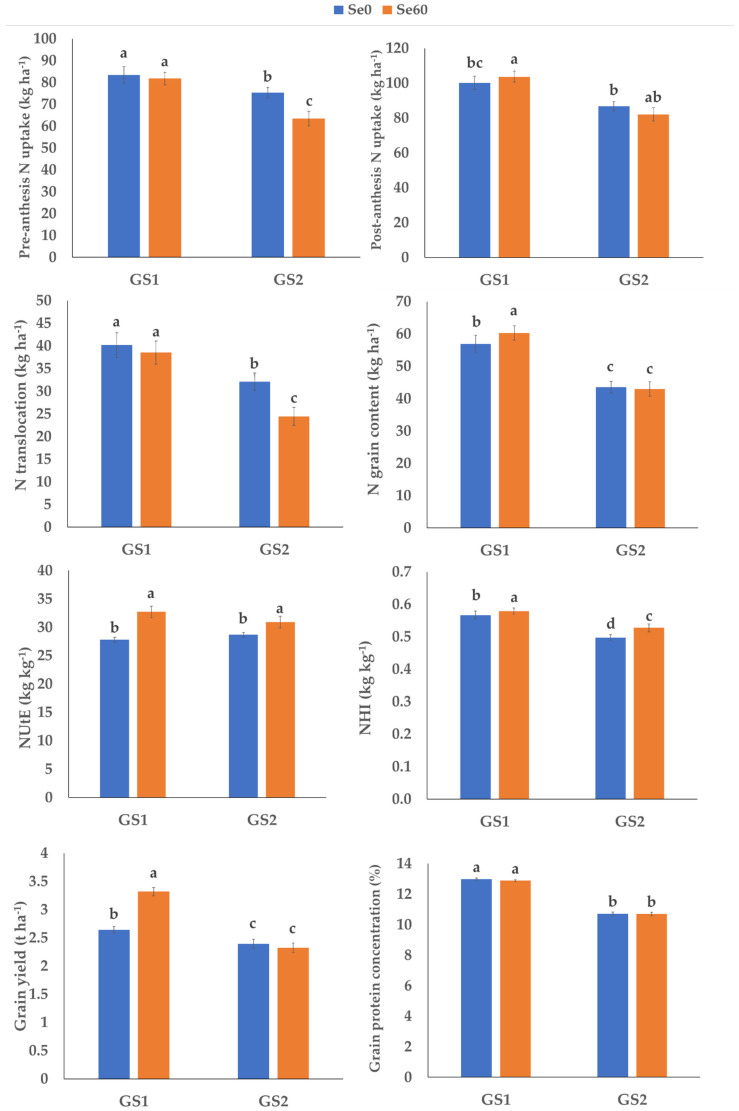
Effect of interaction growing season × selenium application on pre-anthesis N uptake (kg ha^−1^), post-anthesis N uptake (kg ha^−1^), N translocation (kg ha^−1^), nitrogen utilization efficiency (NUtE; kg kg^−1^), nitrogen harvest index (NHI; kg kg^−1^), N grain content (kg ha^−1^), grain yield (t ha^−1^) and grain protein concentration (%). Se0, no selenium application; Se60, selenium application; GS1, 2017–2018 growing season; GS2, 2018–2019 growing season. Letters indicate significant differences at *p* ≤ 0.05 according to Tukey’s test. Vertical bars indicate standard errors (n = 48).

The Se treatment caused an increase in post-anthesis N uptake, NUtE and NHI in both growing seasons, and a decrease in pre-anthesis N uptake and N translocation in GS2. The Se effect was only significant in GS1, when it increased N grain content and grain yield and led to a decrease of grain protein concentration (Figure 2). Se always caused a decrease in pre-anthesis N uptake and N translocation except in Cappelli, and always led to a significant increase in post-anthesis N uptake, expect in Nadif (Table 3). Moreover, Se caused a significant increase in N grain content only in Cappelli. The increase in NUtE under Se treatment was evident in the modern varieties Marco Aurelio and Nadif (Table 3). With regard to NHI, it increased under Se treatment in Cappelli and Nadif and decreased in Marco Aurelio; no Se effect on Old Saragolla was evident. Grain yield increased under Se60 in all varieties excepted in Old Saragolla, while grain protein concentration slightly decreased in the two modern varieties without any significant effect on the old ones (Table 3).

## 3. Discussion

### 3.1. Growing Season × Variety Effects

Since N is a limiting factor in organic farming, the use of varieties capable of efficiently utilizing N is of utmost importance in organic wheat cultivation [1]. Modern varieties with high yield potential are usually indicated as not suitable in organic systems [27]. To gain new experimental data on this subject, we chose to evaluate one landrace (Old Saragolla), one old (Cappelli), and two modern durum wheat varieties (Marco Aurelio and Nadif).

These four varieties differed in their ability to uptake soil N, with large variability in the two growing seasons. The higher capacity of older varieties and landraces to scavenge N deeper pools [28], is a very important aspect in organic farming. In our study this became evident only in the old variety Cappelli which showed the highest pre-anthesis N uptake, especially in GS1, probably due to its well-developed root system [29]. Together with the highest pre-anthesis N uptake, Cappelli showed the highest N translocation value, especially in GS1 where precipitations were higher. This value mostly depends on the N amount stored in the vegetative organs before flowering [30]. The lower soil water availability in GS2 negatively impacted pre-anthesis N uptake, especially in Cappelli and Marco Aurelio, with a consequent decrease in N translocation; indeed, in our experiment these two parameters were highly and positively correlated (*r* = 0.85; *p* ≤ 0.001).

Measurements of post-anthesis N uptake in our experiment also considered foliar N applications at the beginning of the heading stage; for this parameter, an opposite trend with respect to pre-anthesis N uptake was observed in GS1, where modern varieties showed higher values and Cappelli the lowest, with an intermediate behavior of the landrace Old Saragolla. These differences could be attributed to the higher sink strength of modern varieties, that may have enhanced the foliar N adsorption [31]. However, when low rainfall during grain filling occurred, as in GS2, Cappelli showed a marked post-anthesis N uptake increase (more than double than in GS1) while the two modern varieties showed an evident decrease. This could be due to a compensation effect in the drier GS2, when Cappelli was able to increase the post-anthesis N uptake via foliar adsorption. During grain filling, any further N uptake is likely to be allocated directly to the grain [28]. Indeed, we found that post-anthesis N uptake was positively correlated with N grain content (0.43; *p*≤ 0.001) and negatively with N translocation (*r* = −0.43; *p* ≤ 0.001) [32]. For this reason, in the wetter GS1, the modern varieties, which showed the highest post anthesis N uptake, also showed higher N grain content. In the drier GS2, together with the decrease in post-anthesis N uptake, the modern varieties also showed a more evident N grain content decrease (37% for Marco Aurelio and 32% for Nadif) than in Cappelli (22%).

The evaluation of NUE in organic systems is complex and can even be misleading due to the partial unavailability of organic N as fertilizer and also because it is not the only source of available N [1]. For this reason, the evaluation of nitrogen utilization efficiency (NUtE) and nitrogen harvest index (NHI) seems to be more useful in an organic management system. NUtE reflects the ability of the plant to partition N uptake towards grains [28], while NHI reflects the ability to increase grain protein concentration with a given amount of plant N [1]. The higher NUtE and NHI measured on the modern varieties in both growing seasons, as well as the lowest values in Cappelli, confirmed findings from Ortiz-Monasterio et al. [33] on several varieties released between 1950 and 1985 by CIMMYT (Centro Internacional de Mejoramiento de Maíz y Trigo). Elite European winter wheat germplasm has also demonstrated a clear trend towards higher NUtE and NHI over the last 25 years, under high N fertilizations [34]. In this study we demonstrated that better NUtE of the modern varieties is evident also under organic system. In particular, the most interesting differences emerged between the modern Marco Aurelio and the old Cappelli. Under water stress conditions, Cappelli showed a decrease in NUtE, while Marco Aurelio led to a NUtE increase, demonstrating the ability of the latter variety to efficiently use plant nitrogen to make yield even under stress conditions. As a result, yield reduction in GS2 with respect to GS1 was 14% for Marco Aurelio and 32% for Cappelli. Our experiment proved that Marco Aurelio could perform well in organic systems even under water stress conditions, even better than the old variety Cappelli. This was not expected, given that modern varieties are reported to outperform old varieties only in favorable conditions, leading to lower stability across environments and years [35]. This distinct varieties’ behavior in response to climatic fluctuations is crucial in Mediterranean environments, which are characterized by high year-to-year variability of rainfall and temperatures [3] amplified by the ongoing climate change. Moreover, the highest grain yield value showed by Marco Aurelio, which is a high yielding variety [36] was not in agreement with the hypothesis that varieties that perform well under conventional management may not perform well in organic conditions [27]. Accordingly, Kubota et al. [37] studying modern Canadian varieties, reported that they performed relatively well in both conventional and organic management systems considering grain yield and NUtE.

### 3.2. Growing Season × Fertilization Strategy Effects

Winter crops generally suffer significant yield reduction under organic farming due to slow N mineralization during the growth cycle. However, commercial organic fertilizers with a low C/N ratio may lead to higher mineralization, and consequently increase nutrient availability [38]. In this study, the CTR treatment was represented by blood meal (Table S2) with a C/N ratio of 3.03, which can be considered as a rich and quite fast N source [39,40]. Moreover, in our experimental conditions, the frequency and the amount of rainfall in the two growing seasons did not lead to a leaching phenomena, whereas the limited water availability in the second growing season probably caused a lower N mineralization and thus lower N availability for the crop [1]. This was confirmed by the lower pre-anthesis N uptake observed under CTR fertilization in the drier GS2 respect to GS1. Given the evidence that organic fertilizations could limit N availability during grain filling [2,40], we aimed at testing the effect of organic foliar N application at the heading stage on durum wheat. Thus, the pre-anthesis N uptake considers the N uptake by the roots, while the post-anthesis N uptake also considers N absorption from leaves. Surprisingly, the addition of the organic N foliar fertilizer (CTR + N) caused a decrease of pre-anthesis N uptake with respect to the CTR. We hypothesize that the addition of foliar element at the beginning of heading temporarily stopped the root absorption processes. Moreover, in GS1, CTR + N caused a slightly significant increase in NHI without any significant effect on the other parameters, suggesting that in an organic system and under a regular growing season, soil N fertilization with blood meal is a major determining factor for the other parameters [17]. On the contrary, under water stress conditions, foliar N application caused a significant increase in post-anthesis N uptake and N grain content. However, the N accumulated in the grain was not efficiently utilized for the protein synthesis, because it did not cause a positive variation in grain protein concentration with respect to CTR. These results obtained under organic systems are opposite to those obtained in studies conducted under conventional systems where an increase in grain protein concentration after foliar fertilization at anthesis stage has been reported [17,41,42].

Regarding S foliar fertilization, in the wetter GS1, CTR + S led to an increase in post-anthesis N uptake and N grain content with respect to CTR, in agreement with Tea et al., [17] who demonstrated that S applied by foliar spray favored N accumulation in grains. However, the N grain content was not efficiently converted into either protein or yield, since both NUtE and grain protein concentration were lower under S fertilization without any significant effect on yield. On the other hand, also under conventional system, the effect of S on wheat protein concentration and yield is controversial [14,17,43,44,45,46,47,48]. Although several studies have reported a positive effect of S fertilizations under water stress conditions [49,50,51], we found a decrease in pre-anthesis N uptake, N translocation, N grain content, and grain protein concentration in the drier GS2 under CTR + S. On the contrary, the joint N and S foliar fertilization (CTR + NS) increased pre-anthesis N uptake, N translocation, NUtE and grain yield, demonstrating their positive and additive effect [52], under water stress conditions. We can conclude that in our experiment, S and N had a positive and synergic effect only when they were applied as foliar fertilizer at the end of the vegetative stage.

### 3.3. Selenium Effects

Currently the Commission Regulation (EC) No 889/2008 admits the use of trace elements in organic farming. However, to the best of our knowledge, no studies have investigated the effect of selenium on durum wheat under organic management. Selenium beneficial effects on stress tolerance have been frequently reported [53,54]: it improves plant tolerance to drought stress by regulating water status [24], increasing chlorophyll in plant leaves [55], and may also protect plants from fungal infection and invertebrate phloem-feeders [25]. For these reasons, the use of Se in an organic system could be relevant. In this study we applied foliar Se as sodium selenate at the booting stage as suggested by De Vita et al. [56]. The foliar Se treatment caused a decrease in pre-anthesis N uptake only in the drier growing season. Only the old variety Cappelli did not show any variation in pre-anthesis N uptake in relation to the Se treatment, probably due to its robust root system [29]. Due to the high significant correlation between pre-anthesis N uptake and N translocation, also the latter decreased under Se treatment especially in the drier GS2, except in Cappelli.

On the contrary, Se caused a significant increase in post-anthesis N uptake, in both growing seasons and in all varieties, except in Nadif. The increase in post-anthesis N uptake under Se treatment was more evident when N was also added at heading as liquid blood meal and when both foliar N and S were added. Moreover, other studies have reported an increase in N absorption under Se treatment due to a positive influence of selenium on amino acids metabolism [21,57,58,59,60,61,62]. Together with post-anthesis N uptake, Se increased NUtE under all the fertilization strategies applied and especially in the modern varieties Marco Aurelio and Nadif. Under conventional management, several authors reported a positive effect of Se on N assimilation in wheat plants [59,63], probably due to the increase of the nitrate reductase activity [59]. Thus, our experimental evidences referring to organic systems are relevant, since part of the mechanism for the slowing down of the N metabolism under low N availability is due to the reduction in nitrate reductase activity [59] and so, the use of Se in combination with modern varieties evidently seems to improve this relevant aspect.

Foliar Se and N application favored the N accumulation in the grain; however, this increase did not lead to an increase in grain protein concentration. On the contrary, the Se treatment caused a decrease in grain protein concentration in GS1 and in the two modern varieties. Probably, N uptake was not efficiently converted in protein, according to Lara et al. [23] who found N in inorganic forms such as nitrate, nitrite, and ammonium in *Triticum aestivum* grain under Se treatment.

The effect of Se on wheat yield is still controversial. Several authors have reported increases in wheat grain yield after selenate foliar applications [20,21,22,23], while Grant et al. [64], Broadley et al. [65] and Tang et al. [66] reported no significant effect of Se application on yield. In our experimental conditions, we observed a positive effect of Se treatment on grain yield in the first growing season, when higher and well distributed rainfall occurred, leading to a 17% yield increase. Moreover, among the four varieties under study, the Cappelli showed grain yield increase of 17% mainly attributed to post-anthesis N uptake, while modern varieties Marco Aurelio and Nadif showed a yield increase of 13% and 14%, respectively, under Se treatment probably due to their higher NUtE values. The landrace Old Saragolla grain yield was not affected by Se treatment.

## 4. Materials and Methods

### 4.1. Field Trials

Experimental field trials were conducted in two consecutive growing-seasons, 2017–2018 and 2018–2019 (namely GS1 and GS2, respectively), in a field managed according to standard organic farming practices at the Research Centre for Cereal and Industrial Crops (CREA-CI) in Foggia, Southern Italy (41°46′ N, 16°54′ E). Four Italian durum wheat (*Triticum turgidum* ssp. *durum*) varieties, including two old (Old Saragolla, landrace, released in 1900 and Cappelli released in 1915) [67] and two modern (Marco Aurelio and Nadif, released in 2010 and 2016, respectively) were grown on a clay soil (United States Department of Agriculture Classification, Washington, DC, USA) with the following main agrochemical characteristics (in 2018 and 2019, respectively): organic matter (Walkley-Black method) 2.5 and 2.6%; available phosphorus (Olsen method) 62.0 and 68.0 mg kg−1; exchangeable potassium (ammonuim acetate method) 422 and 450 mg kg−1; total nitrogen (Dumas method) 1.3 and 1.1‰. The fields were homogeneous and without preceding crop (set-aside). The sowing date was on 1 December and 24 November 2018 and 2019, respectively, with a seeding rate of 350 germinable seeds m^−2^.

Four organic fertilization managements were evaluated (Table 4): (1) control (CTR) fertilized with dry blood meal in a single application at seeding; (2) CTR plus foliar S application (CTR + S) at flag leaf sheath opening stage (BBCH stage 47); (3) CTR plus N foliar application (CTR + N) at beginning of heading (BBCH stage 51); (4) CRT plus combination of N and S foliar applications at flag leaf sheath opening stage and at the beginning of heading, respectively (CRT + NS). In addition, in all treatments, the selenium (Se) application effect was evaluated by comparing (i) Se0, control without selenium and (ii) Se60, with one foliar application of sodium selenate (Na_2_SeO_4_; BioXtra), at rate of 60 g ha^−1^ [56] at the booting stage (BBCH stage 41) [68]. In each growing season, the experiment was set up in a split–split plot design with three replicates: the variety was the main plot, the organic fertilization management was the plot, and the selenium application was the sub-plot (10.2 square meter).

Foliar fertilizer applications were performed with a hand-held knapsack sprayer utilizing a water solution of bio sulfur (10% *w*/*v*) for CTR + S and a water solution of blood meal (5% *w*/*v*) for CTR + N, without adding the wetting agent. Weeds management during the two growing seasons were performed according to the organic agronomic technique commonly adopted by local farmers, following the Council Regulation (EC) No 834/2007.

Durum wheat grain was machine-harvested at full maturity on 29 and 18 June in 2018 and 2019, respectively, using a Wintersteiger Nursery Master Elite plot combine (Wintersteiger Inc., Ried im Innkreis, Austria).

During the experimental period, the daily rainfall and temperature were recorded by a weather station near the experimental area. According to the Köppen classification, the climate of the experimental site is typically Mediterranean, with De Martonne aridity index indicating a semiarid climate. In general, mean annual rainfall is around 450 mm and mean annual temperature ranges from 12 to 17 °C [69].

**Figure 3:**
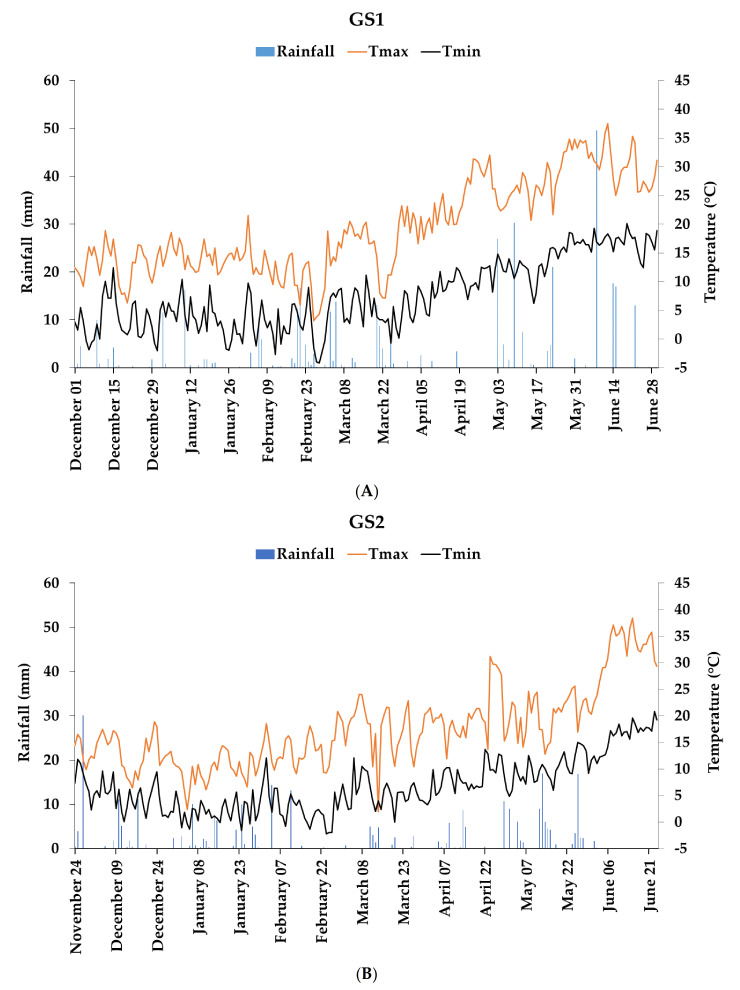
Daily rainfall and maximum and minimum temperatures in the two growing seasons, GS1 (**A**) and GS2 (**B**).

Total rainfall during the crop cycle was 401 mm in the first growing season (GS1; December 2017–June 2018) and 299 mm in the second growing season (GS2; November 2018–June 2019) (Figure 3). Thus, the average rainfall recorded in both growing seasons was below the long-term average, especially in the second growing season. GS1 was characterized by a higher number of days with daily maximum temperature between 30 °C and 35 °C (23 days in 2018 and 6 days in 2019) during the grain filling period, whereas 8 days with air temperature above 35 °C were recorded in GS2 (3 days in GS1).

### 4.2. Yield, Protein Concentration, N-Related Traits, and N Efficiency Indices

At harvest time, the grain yield (t ha^−1^) was assessed for each plot. Grain protein concentration was also determined by near-infrared reflectance spectroscopy, using an Infratec 1229 grain analyzer (Foss Tecator, Hillerød, Denmark).

To evaluate N-related traits (pre-anthesis N uptake, post-anthesis N uptake, N translocation and N grain content), in each experimental growing season, at anthesis (BBCH stage 69) and at physiological maturity (BBCH stage 87) [68], plant samples were taken from 0.5 linear meter of two adjacent rows cutting-off the shoots at the crown level. Moreover, at physiological maturity, plant samples were separated into straw and grain. Dry matter was determined by drying the samples in an oven at 65 °C until constant weight was reached. Subsequently, all samples were ground using a Cyclotec Sample Mill 1093 (Foss Tecator, Hillerød, Denmark). Nitrogen concentration of all samples was determined in triplicate using a Leco CHNS 628 Analyzer (Leco corporation, St. Joseph, Michigan). N content was calculated by multiplying the dry weight by the concentration of N of each sample; the plant’s N content at physiological maturity was the sum of the N content of the straw and grain. Plant N content at anthesis was considered as a measure of the pre-anthesis N uptake, while post-anthesis N uptake was estimated as a difference between plant N content at maturity and plant N content at anthesis [32]. The N translocation during grain filling was calculated following Giuliani et al. [70] as a difference between plant N content at anthesis and straw N content at maturity. We assumed that N was totally translocated from the vegetative part to the developing grain, since losses of N due to volatilization during grain filling were not determined [71,72]. Finally, relative to the N efficiency indices, nitrogen utilization efficiency (NUtE) was calculated as grain yield/plant N content at maturity, and nitrogen harvest index (NHI) as grain N content/plant N content at maturity [37].

### 4.3. Statistical Analysis

The dataset was tested according to the basic assumptions of analysis of variance (ANOVA). The normal distribution of the experimental error and the common variance of the experimental error were verified through Shapiro–Wilk and Levene’s tests, respectively. When required, Box–Cox transformations [73] were applied prior to analysis. ANOVA, derived from linear mixed-effects (lme) model [74], was performed by using the ‘nlme’ package in “R” statistical software, version 3.6.3 [75]. A combined analysis of experimental data over the two growing seasons (GS1 and GS2) was performed. Variety was the whole plot, fertilizer strategy was the split-plot, and Se application was randomized within the split-split-plot. Fixed effects were year, variety, fertilizer strategy and Se application, and random effects were replicates and their interactions with experimental variables. Furthermore, the statistical significance of the difference among the means was determined using Tukey’s honest significance difference post hoc test at the 5% probability level.

## 5. Conclusions

In this study, we demonstrated that modern varieties grown under organic system can lead to better N utilization efficiency with respect to old varieties. In particular, Marco Aurelio had a higher efficiency in using plant nitrogen to increase yield even under stress conditions. The response of this variety to climatic variability is crucial in Mediterranean environments, which are characterized by high year-to-year variability in rainfall and temperature, also in relation to the ongoing climatic changes.

With respect to organic fertilization strategies, we confirmed that soil N fertilization with blood meal at seeding mostly influenced all considered parameters, since the addition of foliar N at heading did not increase neither grain protein concentration nor grain yield in GS1, and even caused their decrease in the dries growing season (GS2).

S and N had a positive and synergic effect, especially under drought conditions, on pre-anthesis N uptake, N translocation, NUtE and grain yield; this was evident only when N and S were applied as foliar fertilizer at the end of the vegetative stage.

We observed a positive effect of Se treatment on post-anthesis N uptake and NUtE, determining also a yield increase of 17% on the old variety Cappelli, mainly attributed to post-anthesis N uptake increase, and of 13% and 14% for the modern varieties Marco Aurelio and Nadif, mainly due to NUtE increase.

In conclusion, this study highlighted the synergistic effect of multiple foliar applications on NUE in organic durum wheat, and their potential to improve production standards even in dry growing seasons. The promising effect of Se foliar application opens new perspectives to improve crop productivity of organic systems, even if further studies must be conducted to validate our experimental findings.

## Figures and Tables

**Table 1:** F-value relative to growing season (GS), variety (V), fertilization strategy (Fs), Selenium application (Se) and their interactions resulting from analysis of variance (ANOVA) performed on pre-anthesis N uptake, post-anthesis N uptake, N translocation, nitrogen utilization efficiency (NUtE), nitrogen harvest index (NHI), N grain content, grain yield and grain protein concentration.

	DF	Pre-Anthesis N Uptake	Post-Anthesis N Uptake	N Translocation	N Grain Content	NUtE	NHI	Grain Yield	Grain Protein Concentration
Growing season (GS)	1	419.7 ***	232.6 **	224.7 ***	527.4 ***	0.7 ns	266.1 ***	569.7 ***	20436.5 ***
Variety (V)	3	237.4 ***	46.2 **	111.8 ***	119.5 ***	243.0 ***	158.0 ***	349.9 ***	2295.3 ***
Fertilization strategies (Fs)	3	53.5 ***	273.9 ***	31.2 ***	4.3 **	1.1 ns	10.6 ***	7.4 ***	112.1 ***
Selenium application (Se)	1	108.7 ***	765.3 ***	71.2 ***	6.6 *	91.4 ***	62.1 ***	161.8 ***	30.2 ***
GS × V	3	79.9 ***	848.1 ***	100.6 ***	60.5 ***	43.9 ***	24.6 ***	18.0 ***	1390.2 ***
GS × Fs	3	160.4 ***	393.3 ***	91.8 ***	72.7 ***	58.3 ***	1.19 ns	11.8 ***	32.4 ***
V × Fs	9	93.0 ***	357.1 ***	47.5 ***	87.9 ***	21.7 ***	10.4 ***	13.8 ***	103.5 ***
GS × Se	1	63.6 ***	20.0 ns	29.3 ***	12.4 ***	12.7 ***	10.0 **	241.8 ***	17.4 ***
V × Se	3	18.8 ***	51.7 **	17.5 ***	28.7 ***	26.6 ***	66.0 ***	8.4 **	6.0 ***
FT × Se	3	24.2 ***	128.1 ***	25.5 ***	19.6 ***	9.4 ***	19.7 ***	15.9 ***	13.8 ***
GS × V × Fs	9	104.6 ***	105.0 ***	79.0 ***	51.7 ***	15.8 ***	17.2 ***	4.6 ***	20.9 ***
GS × V × Se	3	59.8 ***	475.4 ***	54.8 ***	29.6 ***	21.6 ***	54.8 ***	3.4 *	6.2 ***
GS × Fs × Se	3	22.4 ***	559.9 ***	36.0 ***	46.2 ***	5.4 **	65.4 ***	3.1 *	1.1 ns
V × Fs × Se	9	25.7 ***	142.2 ***	33.8 ***	16.6 ***	11.0 ***	30.7 ***	2.9 **	13.9 ***
GS × V × Fs × Se	9	2.5 *	4.2 ***	54.7 ***	231.5 ***	40.5 ***	32.6 ***	20.5 ***	49.0 ***

DF, degree of freedom; *** *p* ≤ 0.001; ** *p* ≤ 0.01; * *p* ≤ 0.05; ns, not significant.

**Table 2:** Effect of interaction growing season × fertilization strategies on pre-anthesis N uptake (kg ha^−1^), post-anthesis N uptake (kg ha^−1^), N translocation (kg ha^−1^), nitrogen utilization efficiency (NUtE; kg kg^−1^), nitrogen harvest index (NHI; kg kg^−1^), N grain content (kg ha^−1^), grain yield (t ha^−1^) and grain protein concentration (%). GS1, 2017–2018 growing season; GS2, 2018–2019 growing season; CTR, control fertilized with dry blood meal at seeding; CTR + N, control plus N foliar application at the beginning of heading stage (BBCH stage 51); CTR + S, control plus S foliar application at flag leaf sheath opening stage (BBCH stage 47); CTR + NS, control plus combination of S and N foliar applications at flag leaf sheath opening stage and at the beginning of heading (BBCH stage 47 and 51), respectively (for more details see Table 4). Values in row followed by different letters are significantly different at *p* ≤ 0.05 according to Tukey’s test. Data are reported as means ± standard errors (n = 24).

Experimental Factor	GS1				GS2			
	CTR	CTR + N	CTR + S	CTR + NS	CTR	CTR + N	CTR + S	CTR + NS
Pre-anthesis N uptake	85.7 ± 4.2 ^ab^	78.8 ± 6.6 ^cd^	88.8 ± 3.5 ^a^	76.8 ± 4.0 ^cd^	76.3 ± 5.5 ^d^	66.8 ± 2.8 ^e^	3.6 ±3.4 ^f^	80.8 ± 2.0 ^bc^
Post-anthesis N uptake	15.71 ± 2.15 ^e^	17.7 ± 2.33 ^b–e^	22.70 ± 4.05 ^abc^	21.27 ± 3.39 ^a–d^	12.75 ± 1.33 ^e^	24.16 ± 4.97 ^a^	16.75 ± 1.75 ^cde^	6.44 ± 0.69 ^f^
N translocation	41.0 ± 0.7 ^a^	38.9 ± 4.0 ^a^	42.2 ± 4.1 ^a^	35.2 ± 3.5 ^bc^	32.2 ± 3.3 ^c^	24.4 ± 2.8 ^d^	19.1 ± 2.1 ^e^	37.3 ± 1.6 ^ab^
NUtE	30.5 ± 1.5 ^bcd^	33.4 ± 1.9 ^abc^	27.3 ± 0.7 ^efg^	29.9 ± 0.9 ^b–f^	29.5 ± 2.6 ^c-f^	25.7 ± 1.7 ^fg^	33.5 ± 1.8 ^ab^	30.5 ± 1.9 ^b–e^
NHI	0.561 ± 0.016 ^bc^	0.591 ± 0.009 ^a^	0.568 ± 0.019 ^b^	0.573 ± 0.014 ^ab^	0.507 ± 0.015 ^de^	0.526 ± 0.017 ^cd^	0.512 ± 0.011 ^de^	0.504 ± 0.018 ^e^
N grain content	56.7 ± 2.5 ^b^	56.6 ± 3.6 ^b^	64.9 ± 4.7 ^a^	56.3 ± 2.2 ^b^	44.9 ± 2.8 ^d^	48.6 ± 3.7 ^c^	35.9 ± 2.1 ^e^	43.8 ± 1.9 ^d^
Grain yield	3.04 ± 0.14 ^a^	2.96 ± 0.10 ^a^	3.01 ± 0.12 ^a^	2.92 ± 0.11 ^a^	2.38 ± 0.10 ^c^	2.20 ± 0.10 ^d^	2.29 ± 0.13 ^cd^	2.56 ± 0.12 ^b^
Grain protein concentration	13.00 ± 0.10 ^a^	13.06 ± 0.11 ^a^	12.68 ± 0.07 ^b^	13.00 ± 0.09 ^a^	10.81 ± 0.19 ^c^	10.72 ± 0.16 ^d^	10.62 ± 0.11 ^e^	10.70 ± 0.15 ^d^

**Table 3:** Effect of interaction selenium application × variety on pre-anthesis N uptake (kg ha^−1^), post-anthesis N uptake (kg ha^−1^), N translocation (kg ha^−1^), nitrogen utilization efficiency (NUtE; kg kg^−1^), nitrogen harvest index (NHI; kg kg^−1^), N grain content (kg ha^−1^), grain yield (t ha^−1^) and grain protein concentration (%). Se0, no selenium application; Se60, selenium application. Values in rows followed by different letters are significantly different at *p* ≤ 0.05 according to Tukey’s test. Data are reported as means ± standard errors (n = 24).

Experimental Factor	Se_0_				Se_60_			
	Cappelli	Old Saragolla	Marco Aurelio	Nadif	Cappelli	Old Saragolla	Marco Aurelio	Nadif
Pre-anthesis N uptake	89.6 ± 5.85 ^a^	68.1 ± 3.57 ^d^	79.8 ± 3.38 ^b^	79.8 ± 4.56 ^b^	88.3 ± 6.19 ^a^	62.7 ± 3.29 ^e^	74.1 ± 3.36 ^c^	65.3 ± 3.86 ^de^
Post-anthesis N uptake	12.0 ± 1.54 ^e^	12.8 ± 1.85 ^de^	14.4 ± 2.00 ^cde^	17.3 ± 2.16 ^bcd^	20.2 ± 5.19 ^ab^	18.6 ± 2.05 ^ab^	23.1 ± 3.61 ^a^	19.0 ± 3.87 ^abc^
N translocation	37.0 ± 3.46 ^ab^	28.4 ± 2.7 ^c^	39.1 ± 2.7 ^a^	40.0 ± 4.3 ^a^	38.7 ± 4.7 ^ab^	22.3 ± 2.0 ^d^	29.9 ± 2.3 ^c^	35.1 ± 3.6 ^b^
NUtE	21.2 ± 0.94 ^e^	30.9 ± 1.73 ^c^	34.3 ± 1.29 ^b^	26.6 ± 1.00 ^d^	23.1 ± 1.47 ^e^	30.7 ± 0.96 ^c^	38.2 ± 1.58 ^a^	35.4 ± 1.53 ^b^
NHI	0.477 ± 0.01 ^e^	0.507 ± 0.01 ^d^	0.566 ± 0.01 ^b^	0.579 ± 0.02 ^b^	0.540 ± 0.02 ^c^	0.505 ± 0.01 ^d^	0.537 ± 0.02 ^c^	0.632 ± 0.01 ^a^
N grain content	48.9 ± 3.66 ^d^	41.3 ± 2.19 ^e^	53.5 ± 2.75 ^bc^	57.3 ± 4.03 ^ab^	58.9 ± 3.88 ^a^	40.9 ± 1.66 ^e^	53.0 ± 3.87 ^cd^	53.8 ± 3.65 ^bc^
Grain yield	2.04 ± 0.056 ^e^	2.37 ± 0.05 ^d^	3.13 ± 0.07 ^b^	2.53 ± 0.08 ^d^	2.38 ± 0.14 ^d^	2.47 ± 0.10 ^d^	3.54 ± 0.11 ^a^	2.89 ± 0.13 ^c^
Grain protein concentration	12.47 ± 0.16 ^a^	12.05 ± 0.25 ^b^	11.52 ± 0.36 ^c^	11.38 ± 0.20 ^d^	12.47 ± 0.17 ^a^	12.01 ± 0.25 ^b^	11.39 ± 0.33 ^d^	11.30 ± 0.18 ^e^

**Table 4:** Levels of organic fertilizer application during field trial. CTR = control; CTR + N = control plus N foliar application; CTR + S = control plus S foliar application; CTR + NS = control plus N and S foliar application.

Organic Fertilizer	Sowing	Flag Leaf Sheath Opening	Beginning of Heading
	Dry Blood Meal (N14.5%; C44%; C/N3.03)	Bio Sulphur (S = 50%)	Liquid Blood Meal (N = 4%)
CTR	50 kg ha^−1^	-	-
CTR + N	50 kg ha^−1^	-	20 kg ha^−1^
CTR + S	50 kg ha^−1^	45 kg ha^−1^	-
CTR + NS	50 kg ha^−1^	45 kg ha^−1^	20 kg ha^−1^

## Data Availability

Data will be made available by the authors upon written request.

## Supplementary Materials

The following are available online at https://www.mdpi.com/article/10.3390/plants10112444/s1, Table S1: F-value relative to growing season (GS), variety (V), fertilization strategy (Fs), Selenium application (Se) and their interactions resulting from analysis of variance (ANOVA) performed on pre-anthesis N uptake, post-anthesis N uptake, N translocation, nitrogen utilization efficiency (NUtE), nitrogen harvest index (NHI), N grain content, grain yield and grain protein concentra-tion, Table S2: Levels of organic fertilizer application during field trial. CTR = control; CTR + N = control plus N foliar application; CTR + S = control plus S foliar application; CTR + NS = control plus N and S foliar application.
