# Supplementary material for: Agronomic Strategies to Improve N Efficiency Indices in Organic Durum Wheat Grown in Mediterranean Area
Source: Plants (Basel). 2021 Nov 12;10(11):2444. doi: 10.3390/plants10112444 (PMC8618784; doi:10.3390/plants10112444)
Supplement: Supplementary file 1 [file plants-10-02444-s001.zip › plants-1437823-supplementary.pdf]

Table S1. F-value relative to growing season (GS), variety (V), fertilization strategy (Fs), Selenium application (Se) and their interactions resulting from analysis of variance (ANOVA) performed on pre-anthesis N uptake, post-anthesis N uptake, N translocation, nitrogen utilization efficiency (NUtE), nitrogen harvest index (NHI), N grain content, grain yield and grain protein concentration.

	**DF**	**Pre-anthesis N uptake**	**Post-anthesis N uptake**	**N translocation**	**N grain content**	**NUtE**	**NHI**	**Grain Yield**	**Grain protein concentration**
**Growing season (GS)**	1	419.7***	232.6**	224.7***	527.4***	0.7 ns	266.1***	569.7***	20436.5***
**Variety (V)**	3	237.4***	46.2**	111.8***	119.5***	243.0***	158.0***	349.9***	2295.3***
**Fertilization strategies (Fs)**	3	53.5***	273.9***	31.2***	4.3**	1.1 ns	10.6***	7.4***	112.1***
**Selenium application (Se)**	1	108.7***	765.3***	71.2***	6.6*	91.4***	62.1***	161.8***	30.2***
**GS x V**	3	79.9***	848.1***	100.6***	60.5***	43.9***	24.6***	18.0***	1390.2***
**GS x Fs**	3	160.4***	393.3***	91.8***	72.7***	58.3***	1.19 ns	11.8***	32.4***
**V x Fs**	9	93.0***	357.1***	47.5***	87.9***	21.7***	10.4***	13.8***	103.5***
**GS x Se**	1	63.6***	20.0 ns	29.3***	12.4***	12.7***	10.0**	241.8***	17.4***
**V x Se**	3	18.8***	51.7**	17.5***	28.7***	26.6***	66.0***	8.4**	6.0***
**FT x Se**	3	24.2***	128.1***	25.5***	19.6***	9.4***	19.7***	15.9***	13.8***
**GS x V x Fs**	9	104.6***	105.0***	79.0***	51.7***	15.8***	17.2***	4.6***	20.9***
**GS x V x Se**	3	59.8***	475.4***	54.8***	29.6***	21.6***	54.8***	3.4*	6.2***
**GS x Fs x Se**	3	22.4***	559.9***	36.0***	46.2***	5.4**	65.4***	3.1*	1.1 ns
**V x Fs x Se**	9	25.7***	142.2***	33.8***	16.6***	11.0***	30.7***	2.9**	13.9***
**GS x V x Fs x Se**	9	2.5*	4.2***	54.7***	231.5***	40.5***	32.6***	20.5***	49.0***

DF, degree of freedom; *** P ≤ 0.001; ** P ≤ 0.01; * P ≤ 0.05; ns, not significant

Table S2. Levels of organic fertilizer application during field trial. CTR = control; CTR+N = control plus N foliar application; CTR+S = control plus S foliar application; CTR+NS = control plus N and S foliar application.

**Organic fertilizer**	Sowing	Flag leaf sheath opening	Beginning of heading
	**Dry blood meal** (N14.5%; C44%; C/N3.03)	**Bio Sulphur** (S=50%)	**Liquid bood meal** (N=4%)
kg ha^{-1}	50	45	20
CTR	✓	✓	
CTR+N	✓		✓
CTR+S	✓	✓	
CTR+NS	✓	✓	✓
